# Biosensors for Point Mutation Detection

**DOI:** 10.3389/fbioe.2021.797831

**Published:** 2021-12-15

**Authors:** Hanlin Jiang, Hui Xi, Mario Juhas, Yang Zhang

**Affiliations:** ^1^ College of Science, Harbin Institute of Technology, Shenzhen, China; ^2^ Medical and Molecular Microbiology Unit, Department of Medicine, Faculty of Science and Medicine, University of Fribourg, Fribourg, Switzerland

**Keywords:** point mutation, detection, biosensor, cancer, virus, SARS – CoV – 2

## 1 Introduction

Point mutations referring to a single base pair change, have a profound effect on the phenotype and play important roles in the variety of diseases (Nasis et al., 2004; Silva et al., 2017; Breveglieri et al., 2018). For instance, antibiotic resistant bacteria, such as carbapenemase-producing Enterobacterales (CPE), including *Klebsiella pneumoniae* represent a major threat to public health. Point mutations in the carbapenemase *bla*_*KPC*_ gene of *K. pneumoniae* were shown to dramatically alter its susceptibility to the whole range of antibiotics (Barnes et al., 2017; Goettig et al., 2019; Poirel et al., 2020). Phosphoinositol-3-kinase, catalytic α-peptide (*PIK3CA*) gene mutation is one of the most common mutations in human cancer and a known biomarker of breast cancer, colorectal cancer, cervical cancer, endometrial cancer, ovarian cancer and other cancer types (Xu et al., 2017a). The *K-ras* gene mutation can be used as an indicator for early diagnosis of lung cancer, colorectal cancer and pancreatic cancer (Gazdar and Virmani, 1998). The *TP53* mutations usually occur more frequently in advanced cancer patients, metastatic disease sites and undifferentiated tumors and can be used to predict chemotherapy or radiotherapy resistance (Shih et al., 2008; Altintas and Tothill, 2012). Detection of point mutations is crucial for screening, predicting and diagnosing tumors. Consequently, the identification and quantification of point mutations has attracted a lot of attention in clinical diagnosis, pathological detection and genetic research (Pinzon-Arteaga et al., 2020).

Although gene sequencing and PCR based methods are the gold standards for point mutation detection, their applications are limited due to the complexity and high costs and instrument requirements. Recently, several new methods for point mutation detection have been developed. These methods are usually based on optical (Zhao et al., 2016), electrical (Zhang et al., 2008) and piezoelectric (Dell'Atti et al., 2006) mechanisms and combined with DNAzyme (Ma et al., 2019), MutS protein (Chen et al., 2009) and isothermal amplification (Xiang et al., 2013) in biosensors. Biosensors overcome many shortcomings of the traditional point mutation detection methods and are therefore a promising tool for the reliable and efficient detection of point mutations.

Furthermore, the application of biosensors for the detection of virus point mutations has been also reported. Reliable and effective detection of point mutations in viruses, such as Severe acute respiratory syndrome coronavirus 2 (SARS-CoV-2) is essential for the control of the epidemics caused by viruses. We discuss the current progress in the point mutation detection approaches, including development of novel biosensors and their potential application in the detection of point mutations.

## 2 Common Point Mutations and Related Diseases

Fast and reliable detection of point mutations which are biomarkers of various diseases is crucial for the selection of effective treatment (Sun et al., 2018; Banerjee et al., 2020).

Cystic fibrosis (CF), β - thalassemia syndrome and sickle cell anemia (SCA) are common diseases caused by point mutations. CF is caused by mutations in the *CFTR* gene. The most common mutation is phenylalanine deletion at codon 508 (CTT), resulting in fibrosis of mucus producing organ channel cells (Bosch and De Boeck, 2016). β - thalassemia syndrome is caused by single nucleotide substitution and small deletion. The common mutation is at codon 39 (CAG-TAG) (Gallagher et al., 2016). SCA is caused by the mutation of GAG-GTG in hemoglobin β - globin chain, resulting in hemolysis and chronic anemia (Delaney et al., 2013).

### 2.1 Point Mutations in Cancer

Point mutations play an important role in carcinogenesis. Kirsten rat sarcoma viral oncogene homolog (*K-ras*) is a kind of mouse sarcoid virus oncogene and an important diagnostic and prognostic indicator of cancer. Point mutations in *K-ras* codons 12 and 13 are very common in different types of cancer. In addition, codons Q61, K617 and A146 are also common *K-ras* mutation sites. Colorectal cancer, lung cancer and leukemia were shown to be closely related to *K-ras* (Cicenas et al., 2017; Jones et al., 2017). BRCA mutations increase the risk of breast cancer, ovarian cancer, prostate cancer, gastric cancer and pancreatic cancer, and have been widely studied as potential prognostic and predictive biomarkers of cancer (Marcus et al., 1996; Farmer et al., 2005). *TP53* encodes tumor suppressor protein involved in cell proliferation and carcinogenesis. The *TP53* gene mutation was more frequent in advanced cancer patients, metastatic disease sites and undifferentiated tumors (Cicenas et al., 2017). Phosphatidylinositol-4,5-diphosphate 3-kinase (PI3K) is one of the key kinases in PI3K/AKT1/MTOR pathway, which plays an important role in the growth and proliferation of tumor cells (Wang et al., 2001). *PIK3CA* mutation is one of the most common mutations in human cancer. It has been identified as a biomarker of breast cancer (Haefliger et al., 2020; Mollon et al., 2020), cervical cancer, endometrial cancer, ovarian cancer and parathyroid adenoma cancer (Milano, 2020; Riccardi et al., 2020). Mutations in exon 9 and 20 of *PIK3CA* are closely related to colorectal cancer (Jin et al., 2020).

### 2.2 Point Mutations in Viral Infection

The accumulation of point mutations in hepatitis B virus (*HBV*) gene may be associated with the development of hepatocellular carcinoma. Mutations can change the replication and virulence of the virus, resulting in persistent infection and severe hepatocyte damage, and eventually lead to the occurrence of hepatocellular carcinoma (Wang et al., 2016). Point mutations in blue tongue virus have been shown to affect the vector competence of this vector-borne virus (van Gennip et al., 2019). Point mutations in the surface glycoproteins of hemorrhagic fever with renal syndrome-causing Hantaan virus enhance their incorporation into recombinant vesicular stomatitis virus (rVSV) particles thus increasing virus infectivity (Slough et al., 2019). Point mutations in the helicase domain of the NS3 protein of dengue virus led to its increased replication and circumvention of the type I interferon response (Silveira et al., 2016). Point mutations in the virus polymerase, spike, matrix genes and ORF5 have been detected recently in the genome of the Middle East respiratory syndrome coronavirus (MERS-CoV) using long-term coronavirus infection model of bat cells. However, none of these mutations with the exception of those in ORF5 disrupted the coding sequences of the respective genes (Banerjee et al., 2020). Another study investigating MERS-CoV strains isolated from hospital patients identified point mutations in the receptor-binding domain (RBD) of viral spike (S) protein in 12 out of 13 investigated strains (Kim et al., 2016). All these MERS-CoV mutations led to reduced affinity of RBD to human CD26 and I529T mutation led to reduced entry into host cells (Kim et al., 2016). Two point mutations (N15A and V25F) in the transmembrane domain (TMD) of the envelope (E) protein of the Severe acute respiratory syndrome coronavirus (SARS-CoV) led to virus attenuation *in vivo* (To et al., 2017). Another point mutation (at the valine 68 residue of M protein) in the SARS-CoV genome led to reduction of the virus-induced IFN-β production (Wang and Liu, 2016). Furthermore, single amino acid substitution in the RBD (R441A) suppressed the immunogenicity of RBD in mice and rabbits (He et al., 2006).

## 3 Traditional Point Mutation Detection Methods

The conventional point mutation detection methods include sequencing and PCR based methods (Manam and Nichols, 1991; Riahi et al., 2015). The basic mutation site recognition strategies include surface ligation reaction, mismatch binding protein mediated strategy, molecular beacon-based method and fluorescence *in situ* hybridization (FISH) (Sun et al., 2018; Lu et al., 2020). Tang et al. (Tang et al., 2017) developed a new fluorescence *in situ* hybridization (FISH) strategy combined with a new branch hybridization chain reaction (bHCR) for efficient signal amplification of ligases and detection of specific mutations.

### 3.1 Detection Methods Based on Gene Sequencing

Gene sequencing is the gold standard for detecting point mutations, which has been widely used in various genotyping methods. However, in addition to being laborious and time-consuming, it has low sensitivity and high technical requirements (Millat et al., 2014; Sun et al., 2018).

### 3.2 Detection Methods Based on PCR

PCR based methods, such as PCR based allele detection system (AS-PCR) and fluorescent gene detection technology, allele specific small groove binding probe real-time PCR detection (RT-PCR), PCR restriction fragment length polymorphism (RFLP), polymerase chain reaction single strand conformation polymorphism (PCR-SSCP) and heteroduplex mobility analysis (HMA) belong among the most commonly used point mutation detection methods (Callens and De Clercq, 1999; Pozzi et al., 2009; Riahi et al., 2015; Prykhozhij et al., 2018).

RT-PCR is widely used to improve the sensitivity and timeliness of PCR based detection. RT-PCR has been used for the detection of RNA viruses in infected tissue culture medium by optimizing micro hole hybridization and colorimetry (Callens and De Clercq, 1999). RT-PCR can detect and quantify mutations rapidly and simultaneously. This method has low detection limit and requires no post-processing of PCR. Furthermore, it showed a good correlation with the results of allele specific oligonucleotide hybridization (Singh et al., 2006). ARMS (amplification refractory mutation system)-PCR is a simple and economical technique for mutation detection. ARMS was used to detect the mutations in *katG* and *fabG* genes for rapid diagnosis of *Mycobacterium tuberculosis* (Mtb) drug resistance (Riahi et al., 2015). Real time quantitative PCR (qPCR) was used to detect and measure the 3243A > G mutation in patients with diabetes (Singh et al., 2006). Mahmoudi et al. (Mahmoudi et al., 2019) studied the detection of TR34/L98H mutation in *cyp51A* gene of *Aspergillus fumigatus* by tetra-primer ARMS. The method was optimized by tetra-primer in one reaction, which consisted of an external primer for detecting tandem repeats in the promoter region and an internal primer for detecting point mutation in codon 98 (L98H) of *cyp51A* gene of *Aspergillus fumigatus*. Although this method is simple and low-cost, it is rarely used because of the restriction of primer design and polymorphism region. Shokrani et al. (Shokrani et al., 2012) developed a new PCR-RFLP technique based on an improved forward primer to identify SNPs at codons 167 and 200 of isotype 1 *β-Tubulin* gene. PCR-RFLP is a sensitive but a radioactive method. PCR-SSCP detected the mutation by electrophoretic mobility shift of single stranded DNA in non-denatured polyacrylamide gel. HMA is a highly sensitive and fast analysis method, which can detect all mutations, but it is complex and laborious (Temesgen et al., 1997). The advantages of the PCR based gene sequencing include high specificity and sensitivity and automation, but the method is cumbersome and time-consuming.

### 3.3 Detection Methods Based on Nuclease-Assisted Probe System

The basic principle of the nuclease-assisted probe system to detect point mutation is to design a nucleic acid probe that complements the mutation sequence. The mutation sequence hybridizes with the nucleic acid probe to form double-stranded DNA, which can be specifically recognized and cleaved by nuclease. The wild-type sequence and nucleic acid probe cannot be recognized and cleaved by nuclease. The point mutations can be quantitatively analyzed according to the signal difference before and after cleavage of the target sequence (Xu et al., 2017b; Ming et al., 2021). However, the commonly used nucleases (endonuclease IV, apurinic/apyrimidinic endonuclease-1 (APE-1), etc.) are easily interfered by the surrounding nucleic acids in the system, thus leading to non-specific cleavage (Kulcsár et al., 2017), which increases the background signal and causes errors (Ma et al., 2015). In order to solve this problem, Ming et al. (Ming et al., 2021) designed a new type of nuclease-assisted fluorescent probe system to detect point mutations. The system designed two guide chains and probe sequences modified by carboxyfluorescein (FAM) and black hole quencher (BHQ) at both ends, respectively. The two guide chains complement the regions outside the probe hybrid domain of the target chain, and the target chain, the probe and the two guide chains form a double-stranded structure that can be digested by restriction endonuclease. The mutation sequence can be quantitatively analyzed according to the change of fluorescence intensity after the addition of nuclease. The guide chain and the target chain complement each other and direct the nuclease to the target double-stranded DNA, thus promoting cleavage. The guide chain not only maintained the high cleavage efficiency of nuclease to the target chain, but also significantly inhibited the non-specific cleavage of single strand probe by nuclease.

Sensitive and specific DNA hybridization is the key to detect point mutation in nuclease-assisted probe system. The competitive combination of DNA probe and blocking chain has become the most commonly used probe design because of its relatively high sensitivity and specificity. The blocking chain should be hybridized with wild-type DNA to prevent it from binding to the probe and reduce the percentage of wild-type DNA in the sample, which is beneficial to the subsequent probe recognition process (Smith et al., 2011; Chen et al., 2019). However, the sensitivity and specificity of DNA hybridization negatively correlate with the length and concentration of the blocking chain, which makes the optimization of DNA hybridization more complicated (Chen et al., 2019). Chen et al. (Chen et al., 2019) invented a novel probe/blocker system based on Holliday junction branch migration: the 4-way Strand Exchange LEd Competitive DNA Testing system (4-way SELECT system). The system broke the inherent negative correlation: with the increase of the length and concentration of the blocker chain, the sensitivity remained unchanged, while the specificity increased monotonously until it reached the theoretical maximum. Therefore, the sequence design and reaction conditions of DNA probe can be simplified and unified without any optimization, which greatly facilitates and broadens the application range of DNA probe, especially in high-throughput multi-mutation analysis. The nuclease-assisted probe system can detect low abundance point mutations, but the system usually shows strong sequence dependence and poor specificity for some types of point mutations, therefore it is still a challenge for high-precision detection of point mutations (Yu et al., 2016).

### 3.4 Other Detection Methods

The main limitations of PCR based methods, DNA hybridization and sequencing, are the high costs and instrument requirements. High throughput sequencing includes denaturing high performance liquid chromatography, mass spectrometry and high temperature quenching. These approaches require sample pretreatment, complex processing steps, and complex instruments such as HPLC, laser scanner, mass spectrometry, thermocycles, fluorometer, capillary gel electrophoresis or photometer (Sun et al., 2018; Lu et al., 2020). Other methods are mainly based on allele specific hybridization or enzyme assisted allele recognition, such as enzyme digestion, oligonucleotide ligation and DNA polymerase mediated primer extension. Although the hybridization-based methods are relatively simple, they need complex labeling process and strict control of hybridization conditions. The mismatch recognition of enzymatic reactions is attractive due to its high specificity, easy operation and rapid detection. However, these methods require radioactive or fluorescent labels.

Consequently, cost-effective devices, such as biosensors represent an attractive approach for rapid and large-scale point mutation screening.

## 4 Biosensors for Point Mutation Detection

**FIGURE 1:**
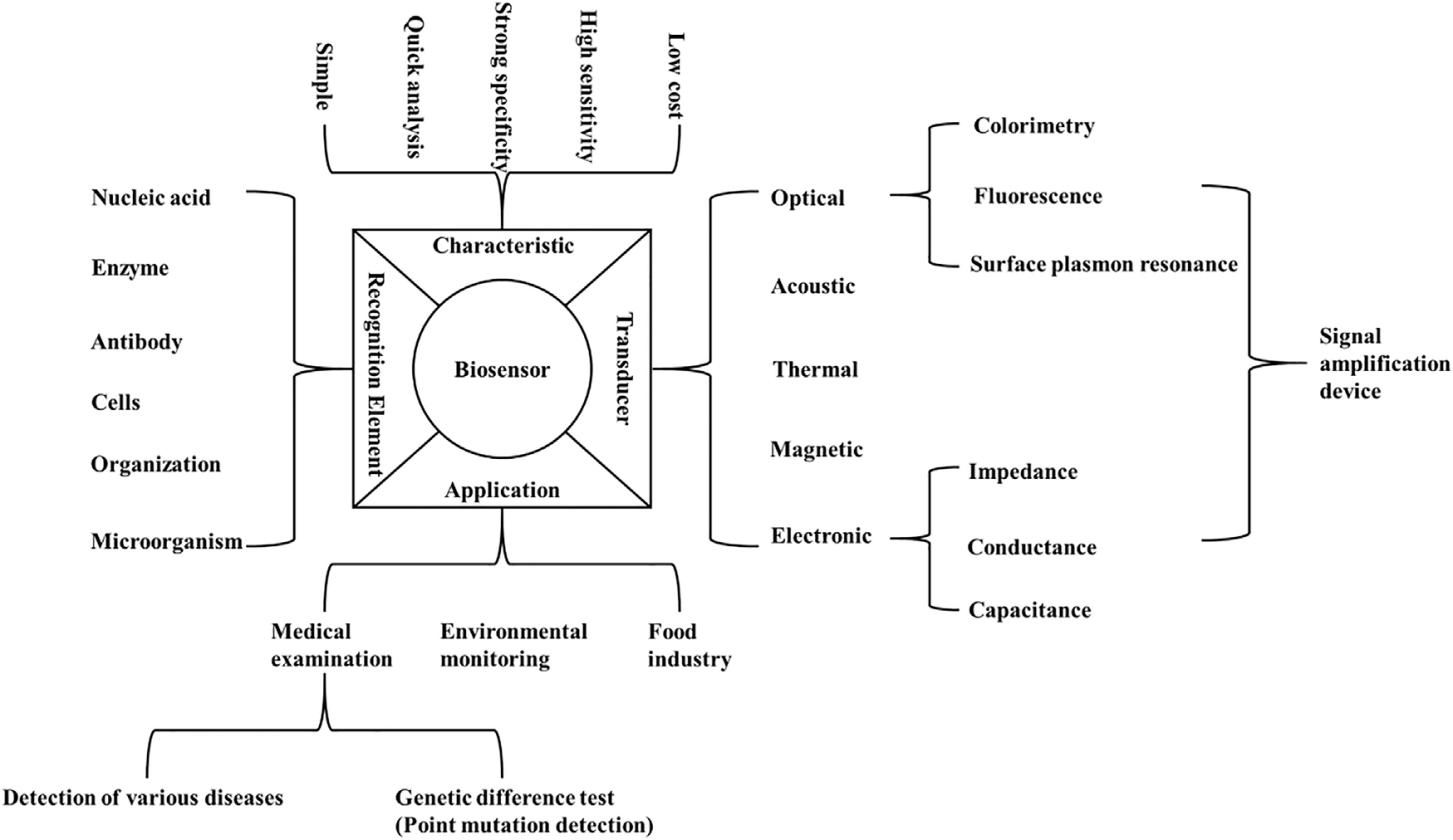
Composition, characteristics and application of biosensor. The figure summarizes the characteristics, recognition elements, applications and transducers of the biosensors which can be used for the detection of point mutations.

A biosensor is an analytical device with high sensitivity, good specificity and low cost. Biosensors are composed of molecular recognition elements and signal converters, which transform biological or chemical reactions into detectable signal outputs (Shlyahovsky et al., 2007; Myszyka, 1999). The biosensor-based methods can overcome the shortcomings of traditional methods in detecting point mutations (Mahmoudi et al., 2019) (Figure 1). In general, the biorecognition elements for nucleic acid detection are ssDNA probes, which hybridize with complementary known ssDNA. These probes are fixed on the surface of the transducer, thus making the DNA hybridization event a measurable electrical signal (Zhang et al., 2008). Based on the signal converters, biosensors can be divided into electronic biosensors, optical biosensors (Zhao et al., 2016), piezoelectric biosensors (Dell'Atti et al., 2006) and other biosensors (Zhu et al., 2009). Biosensors based on electrochemical, fluorescence, surface plasmon resonance (SPR), piezoelectric and electrochemiluminescence (ECL) techniques monitor the hybridization reaction between the probe fixed on the sensing surface and the complementary or mismatched sequence in the solution (Zhu et al., 2009; Li et al., 2014; Cui et al., 2017). Due to the high selectivity, sensitivity and speed, biosensor technology can be used to detect point mutations, thus allowing to reliably detect and monitor variety of diseases (Lu et al., 2020).

### 4.1 Electronic Biosensors

Point mutations are most commonly detected by electronic biosensors. In electronic biosensors the probe is fixed on the surface of the electrode, and the target sequence is captured on the surface of the electrode by a specific action (Zhang et al., 2008). DNA can be hybridized according to the redox characteristics of the base, adding electroactive hybridization indicator to modify the electrode or labeling enzymes (Liu et al., 2020), electroactive groups and nanoparticles (Shoja et al., 2018) as reporters of electrochemical activity. Hybridization is used to detect the changes in electrical signal. For target detection, the electrode converts the concentration signal into measurable electrical response signals such as potential (Zheng et al., 2004), resistance (Duan et al., 2013), current (Song et al., 2006) and capacitance (Guiducci et al., 2004). The electrochemical detection platform meets the requirements of accuracy and real-time detection for reliable gene diagnosis (Lu et al., 2020).

DNA bases will undergo single electron oxidation under a certain potential. Due to the hybridization reaction, the redox site is in the double strand, which reduces the contact sites between DNA and the current surface and reduces the redox current signal (Liu et al., 2010). Due to the low oxidation potential of guanine, differential pulse voltammetry (DPV) has been used to monitor the oxidation signal of guanine and detect the hybridization between the probe and mutant or wild-type DNA (Esteban-Fernández de Ávila et al., 2015). To improve the sensitivity of biosensor dot active hybridization indicator can be added or electrode or probe can be modified.

Raoof et al. (Raoof et al., 2011) developed an electrochemical biosensor based on self-assembled monolayer (SAM) forming thiol peptide nucleic acid (PNA) molecule to prepare probe modified gold electrode. This biosensor has been used to detect the point mutation of *TP53* tumor suppressor gene. The PNA capture probe eliminates the electrostatic repulsion between the two hybrid chains due to the uncharged characteristics. PNA-DNA double stranded body showed more stability than the corresponding DNA-DNA double stranded body. PNA oligomers can invade DNA double stranded bodies in a highly sequence specific manner. A DNA biosensor mediated by methylene blue (MB) was developed by using PNA probe. The hybridization events between PNA probe and DNA were determined by DPV and led to the efficient identification of point mutations. The detection limit of this biosensor is 6.82 × 10^–10^ M. Esteban-Fernández de Ávila et al. (Esteban-Fernández de Ávila et al., 2015) developed an electrochemical biosensor for detecting point mutations in *TP53* gene sequence. Two kinds of specific hairpin capture probes were immobilized on screen printed electrodes (SPCEs) modified with reduced graphene oxide carboxymethyl cellulose (rGO-CMC). Streptavidin peroxidase (Strep-HRP) conjugate was used as an electrochemical indicator. The hybridization current was monitored by adding 3,3′, 5,5′ - tetramethylbenzidine (TMB) as redox medium and H_2_O_2_ as enzyme substrate.

Nano materials have the characteristics of quantum, surface and macroscopic quantum tunneling effects. Functionalized nanoparticles, such as noble metal nanoparticles, carbon nanomaterials and quantum dots, are used as probe markers for DNA biosensors, which improves the sensitivity of target sequence detection and reduce the background signal (Xu et al., 2009). Shoja et al. (Shoja et al., 2018) first reported that EGFR extron 21 L858R mutation which can be used as a common biomarker of lung cancer. The modified ssDNA capture probe immobilized on pencil graphite electrode was modified with reduced graphene oxide (rGO)/functionalized ordered mesoporous carbon/nickel oxytetracycline metal polymer nanoparticles, in which nickel oxytetracycline metal polymer nanoparticles are an electroactive marker. This electrochemical biosensor has good sensitivity, high reproducibility, selectivity and stability. Krejcova et al. (Krejcova et al., 2013) determined the target molecules labeled with quantum dots (QDs) by voltammetry. A magnetic electrochemical bar code array was developed to detect single point mutations (up to four nucleotide mismatches) in the H5N1 neuraminidase gene based on hybridization detection with improved paramagnetic particles automatic separation.

**FIGURE 2:**
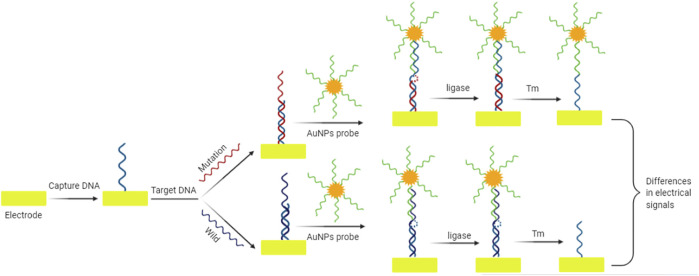
Schematic diagram of electrochemical sensor for mutation detection based on amplification effect of *E. coli* DNA ligase and AuNPs. The gap between the capture probe and AuNPs probe is closed by *E. coli* DNA ligase only in the sequence harboring mutation. The resulting differences in electrical signals can be used for the mutation identification.

High sensitivity is an important feature of biosensors. To further improve the sensitivity of low abundance target detection, a variety of nucleic acid based signal amplification strategies have been applied in electronic biosensors, including enzyme assisted amplification strategies, such as exonuclease III (Exo III) assisted target recovery strategy, rolling cycle amplification (RCA), nicking enzyme signal amplification (NESA) and chain displacement amplification (SDA). For example, Wang et al. (Wang et al., 2020) proposed an effective electrochemical biosensor based on restriction enzyme mediated strand displacement amplification (NSBI-SDA) reaction and four-way DNA linkage for the detection of *PIK3CA* gene mutation. In the presence of target mutant genes, NSBI restriction endonuclease can recognize specific mutation sites and cut dsDNA. The resulting DNA fragments can trigger SDA reaction to generate a large number of ligation chains. SDA reaction is an effective method for target cycle amplification, which can effectively obtain DNA copy and amplify biological signals, recognize *PIK3CA* ^*H107R*^ gene mutation and amplify biological signals. When the linker is trapped on the electrode, the four-way DNA binds to the end of the linker. The signal change was determined by the electroactive molecule of methylene blue (MB). The sandwich electrochemical biosensor can detect target mutation efficiently. Liu et al. (Liu et al., 2020) proposed a novel electrochemical biosensor based on the heat-resistant ligase chain reaction (LCR), which has high point mutation recognition ability. The dsDNA amplified by LCR was evenly distributed on the BSA modified gold electrode for self-assembly. Specific binding and catalytic activity of Strep-HRP were collected on the substrate of TMB containing H_2_O_2_. This biosensor allowed to distinguish the homozygous mutant from the wild-type CYP2C19 allele in the human whole blood samples. LCR is a useful technique for allelic identification, and its amplification efficiency is equivalent to that of PCR. Zhao et al. (Zhao et al., 2016) reported a new method for amplification of *K-ras* point mutation based on *E. coli* DNA ligase and amplification effect of AuNPs. This method allowed to distinguish *K-ras* mutant DNA from *K-ras* wild type. The charge variable (Q) was proportional to the logarithm of *K-ras* mutant DNA concentration ranging from 1.0 nM to 0.1 pM, and the detection limit was 0.01 pM. The use of *E. coli* DNA ligase and AuNPs improves the sensitivity and specificity of detection, and also provides the advantage of low cost and portability for this detection system. After denaturation at high temperature, the wild target DNA of *K-ras* did not match the capture probe and AuNPs probe completely. *E. coli* DNA ligase could not close the gap between capture probe and AuNPs probe, thus only the capture probe remained on the electrode surface. The mutant target DNA of *K-ras* perfectly matched the capture probe and AuNPs probe. Due to the presence of *E. coli* DNA ligase, the gap between the two probes was closed, thus leaving both the capture probe and the AuNPs probe on the electrode surface (Figure 2). Zhang et al. (Zhang et al., 2008) proposed a highly sensitive electrochemical method for point mutation detection based on surface enzyme linking reaction and biometallization. The specific probe fixed on the electrode surface complements the mutation target. In the presence of mutant oligonucleotide target and *E. coli* DNA ligase, biotinylated probe was hybridized with ligation products. By combining streptavidin alkaline phosphatase (SA-ALP) with biotinylated probe, ascorbic acid 2-phosphate (AA-P), the non-reducing substrate of alkaline phosphatase, can be converted to ascorbic acid (AA) on the electrode surface. Silver ions in the solution are reduced by AA, resulting in silver metal deposition on the electrode surface. Linear sweep voltammetry (LSV) was used to detect the single base mutation in codon 12 of *K-ras* oncogene.

Although in these signal amplification strategies the enzymes with high catalytic activity significantly improve sensitivity of detection, their use is limited due to the high price and low anti-interference ability to the external environment (e.g. temperature, pH, ion concentration) (Umek et al., 2000; Wang et al., 2001). DNAzymes with the help of cofactors exhibit catalytic activity for specific substrates, with the advantage of high stability, low cost, easy modification and multiple denaturation and refolding without the loss of catalytic activity. For example, Zhou et al. (Zhou et al., 2020) developed an efficient, enzyme-free and label free *K-ras* G12D point mutation electrochemical sensor using DNAzyme and hybrid chain reaction (HCR) technology. In this electrochemical sensor DNAzyme and HCR are used to amplify the signal. The presence of target induces the cleavage of DNAzyme into a complete active conformation, which catalyzes the cyclic cleavage of the substrate and produces a large number of intermediate products. The intermediate product triggers the downstream HCR to form dsDNA. A large amount of MB is coupled by *π* - *π* stacking, and then two-stage amplification is performed to produce significant electrochemical signals. Under the optimal conditions, the biosensor platform achieves high sensitivity target detection with the minimum detection limit of 0.5 fM.

Biosensors have not only high sensitivity but also high specificity, and can be used to detect mutant DNA mixed with high concentration of wild type DNA. Attoye et al. (Attoye et al., 2020) presented a method that combines a screen-printed carbon electrode and a DNA amplification reaction to specifically detect KRAS G12D mutations. This method can effectively detect mutations in samples with KRAS G12D mutation content of about four copies/ng in the presence of high-background wild type KRAS DNA sequences after just 20 PCR cycles. In this method, mutant probes and wild type probes were designed for KRAS G12D mutant sequence and KRAS wild type sequence, and fixed on the carbon electrode. At the same time, primers were designed to specifically amplify the KRAS G12D mutation sequence or wild type KRAS sequence. The samples were subjected to PCR amplification, and wild type probes and mutant probes were used to hybridize the amplified products. The KRAS G12D mutant sequence and the KRAS wild type sequence could be distinguished based on the changes in the peak current of the cyclic voltammetry curve before and after the hybridization reaction. If the probe hybridizes with the amplified product to form a double strand, the peak current of the cyclic voltammetry curve will decrease; otherwise, the peak current will not change significantly. In recent years, electrochemical biosensors have been widely used to detect point mutations with high sensitivity and specificity (Zhou et al., 2020).

### 4.2 Optical Biosensors

Optical DNA biosensors use the combination of target and probe to change the optical signal. Based on the change of fluorescence, color and refractive index, they can be divided into fluorescence biosensors, colorimetric biosensors and surface plasmon resonance biosensors (Gotoh et al., 1997; Hossain et al., 2020).

#### 4.2.1 Colorimetric Biosensors

Colorimetric biosensors have attracted much attention due to their low cost, simple operation, fast response and good reproducibility. However, their sensitivity is relatively low and requires signal amplification. Oh et al. (Oh and Lee, 2011) rapidly and accurately detected the single base mutation of breast cancer gene by DNA-AuNPs colorimetric detection system based on hybridization characteristics. The effects of ion strength, temperature, time and DNA loading on the chemical hybridization characteristics of DNA-AuNPs were studied. Bai et al. (Bai et al., 2020) designed a hypersensitive colorimetric biosensor for the detection of BRCA1 mutation based on multiple signal amplification strategy. The signal probe was immobilized on the surface of nano materials (AuNPs/Bi_2_Se_3_) to form a signal unit, which could catalyze the reduction of 4-Nitrophenol (4-NP), and the solution changed from bright yellow to colorless. The detection limit of this biosensor is 10^−18^ M. In the linear range, there is a good linear relationship between the reaction kinetic constant and the DNA concentration. In addition, the biosensor can clearly distinguish single base mismatch, double base mismatch and non-complementary sequence. Valentini et al. (Valentini et al., 2013) described a colorimetric biosensor based on gold nanoparticles for cancer-related *K-ras* point mutation. The colorimetric method avoids the use of large-scale instruments and is simple and convenient.

#### 4.2.2 Fluorescent Biosensors

In addition to colorimetric biosensor, fluorescent biosensor is also a common signal reading method for point mutation detection. The phenomenon of “turn off” or “turn on” occurs when molecules with fluorescence signal group in biosensor system are detected. It leads to the change of fluorescence signal and target detection Nanoclusters show strong and size dependent fluorescence emission. They have been developed as a new class of fluorescent group, which can be used as sensors for detecting point mutations (Han et al., 2019). Because the formation of AuNPs is highly sequence dependent, the single stranded nucleotides of inserted cytosine ring were extended to dsDNA to produce fluorescence (Deng et al., 2007). This biosensor can recognize the typical single nucleotide mutation - sickle cell anemia gene mutation. At present, this strategy has been extended to cover the general types of SNPs. Qiu et al. (Qiu et al., 2013) designed a novel DNA sequence fluorescence biosensor based on the cycloaddition reaction of azine catalyzed by copper nanoparticles (CuNPs) and Cu (I) on the dsDNA template. CuNPs induced ‘Copper-Catalyzed Alkyene-Azide Cycloaddition’ reaction between weak fluorescent compounds (3-azido-7-hydroxycoumarin) and propargyl alcohol to form strong fluorescence compounds. Because CuNPs were effectively accumulated in the main channel of dsDNA, while ssDNA had no channel, it showed that the sensor had the advantages of low detection limit, high sensitivity and good selectivity for the detection of mutant *TP53* DNA sequence *in vitro* and *in vivo*.

With the continuous improvement of quantum dot (QD) technology, QDs have been widely studied as fluorescent labeling of biological probes in biosensors. QDs are not only a high-intensity fluorescent label, but also a nano center, which is used to capture multiple conjugated dye products for signal amplification, which is conducive to highly accurate fluorescence detection. Tang et al. (Tang et al., 2015) designed a QD based biosensor for DNA point mutation detection. The biotinylated probe can capture the mutation target of PCR amplification. The biotinylated probe labeled with Cy5 can be further assembled on the surface of QD to obtain Cy5-DNA quantum dot complex, which can produce fluorescence resonance energy transfer (FRET) between QD donor and Cy5 receptor. This biosensor can detect mutation target with high sensitivity with the detection limit of 5.3 aM, and can even discriminate as low as 0.01% variant frequency from the mixture of mutant and wild-type targets. Song et al. (Song et al., 2013) used gap ligase chain reaction (gap-LCR) to generate mutation specific junction products, which were captured by QDs to form DNA-QD nanocomposites. The mutants were identified by multicolor fluorescence quenching single molecule spectroscopy (SMS), which allowed multiple mutation detection in the form of no separation. *K-ras* mutation was successfully detected in the original genomic DNA without PCR pre-amplification. In addition, compounds with aggregation induced luminescence can also be used as fluorescent markers to detect point mutations.

#### 4.2.3 Surface Plasmon Resonance (SPR) Biosensors

**FIGURE 3:**
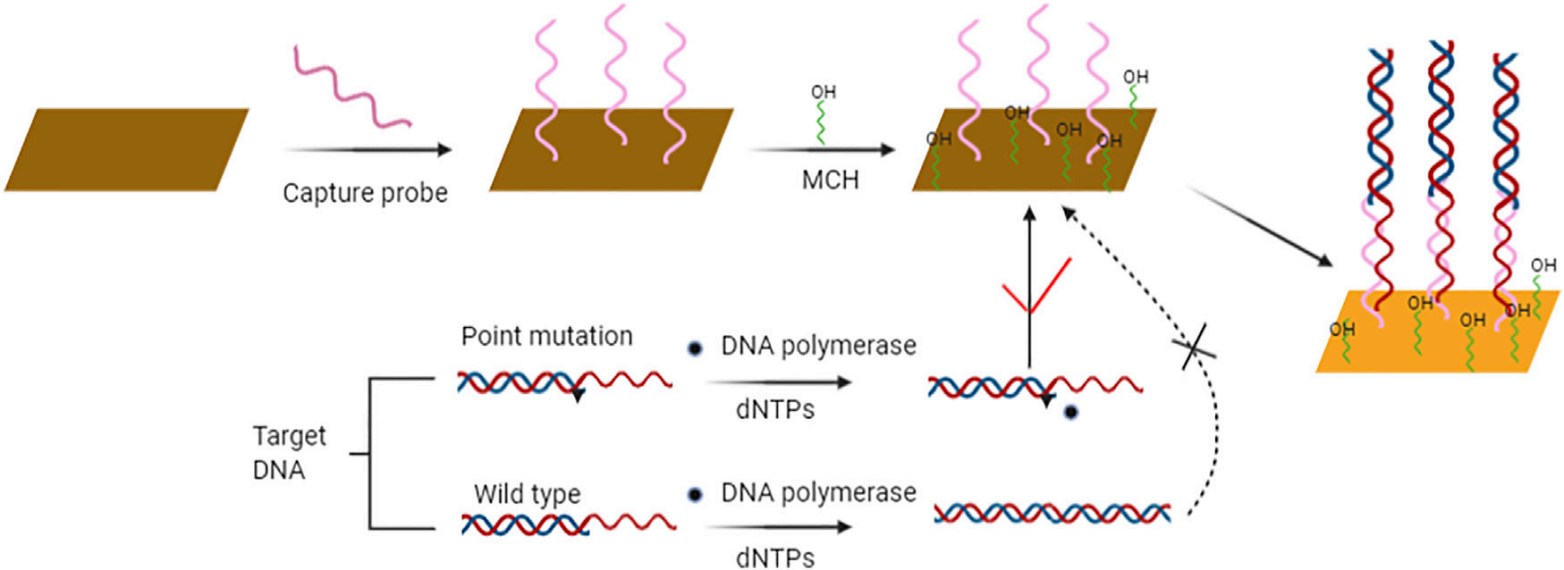
Schematic diagram of SPR biosensor strategy for rapid and sensitive point mutation detection. Contrary to wild type sequence, sequences containing point mutation are not elongated and consequently hybridize with the capture probe on the sensor’s surface and produce measurable signal output.

The sensitivity and specificity of SPR biosensors have been greatly improved over the last years. The main improvements include using peptide nucleic acid as recognition element, DNA modified gold nanoparticles for sandwich analysis, and enzyme reaction for mismatch recognition. Li et al. (Li et al., 2014) constructed a label free and highly sensitive SPR biosensor for point mutation detection based on polymerization extension reaction. The 3′-mercaptan DNA probe with complementary sequence was immobilized on the surface of the sensor by molecular self-assembly. In the presence of wild target sequences, primers can be selectively extended by DNA polymerase to form dsDNA. On the contrary, the mutation target sequence containing a mutation site that does not match the 3′ terminal base of the primer cannot be extended. The product of elongation reaction can be hybridized with the capture probe modified on the surface of the sensor to induce SPR signal (Figure 3). This method can detect *BRCA1* gene mutations associated with hereditary breast cancer, with the detection limit of 100 pM. Gotoh et al. (Gotoh et al., 1997) introduced a new method to detect DNA point mutation by mismatch binding protein. Biosensors based on SPR are used to detect mismatch and mismatch binding protein interactions. Firstly, oligonucleotides are immobilized on the biosensor, and then oligonucleotides containing complementary sequences or single mismatches are applied to allow annealing to form double stranded oligonucleotides. Point mutations are detected in a short time by SPR technology. In SPR biosensor, the concentration, flow rate and temperature of the hybrid buffer have effects on the signal response. Milkani et al. (Milkani et al., 2010) modified SPR biosensor by self-assembly technology. The change of SPR signal is always greater when the mismatch is located in the middle or near end of the target DNA. By comparing the surface hybridization efficiency of proximal, distal and intermediate mismatches, the effects of three hybridization parameters on the detection of single nucleotide mismatch by SPR were studied. Lee et al. (Lee et al., 2005) studied enzymatic amplification of surface plasmon resonance imaging and detection of DNA sequence by Exo III digestion DNA microarray. Enzyme amplification technology can be combined with biosensors with different conversion signals to detect point mutation. Electrical and optical biosensors can be combined with amplification technology to improve the detection sensitivity.

### 4.3 Other Biosensors

DNA based piezoelectric biosensors are also used to detect point mutations. The piezoelectric signal conversion can be detected only by the frequency change of the interface after adding the target. Dell’atti et al. (Dell'Atti et al., 2006) developed a DNA based piezoelectric biosensor for detecting gene mutations at codon 248 of *TP53*. Feng et al. (Feng et al., 2007) combined DNA enzyme-based ligation reaction with quartz crystal microbalance (QCM) measurement to detect point mutations in DNA targets. In this study they used streptavidin peroxidase horseradish conjugate mediated insoluble product of 3,3-diaminobenzidine (DAB) deposited on the electrode carrier as a signal amplification pathway for quantitative detection of target genes. This biosensor combining high specificity of DNA ligase and low cost of QCM for SNPs detection has been successfully applied for the identification of single base mutations in thalassemia gene. It combines the high-fidelity complete matching ligation of *E. coli* DNA ligase with QCM surface biocatalytic precipitation amplification method to carry out microscopic quantitative analysis of the target gene. Based on a similar principle, a number of various ligase-based SNPs detection methods have been developed.

Interesting area of biosensor research focuses at acoustic biosensors. Acoustic biosensors (the QCM) based on monitoring of the length of DNA amplicons have been used for the detection of the three different bacterial phytopathogens (Papadakis et al., 2015). Genetically encoded acoustic biosensors have been shown to be applicable for the visualization of biomolecular activity of enzymes in deep tissues of living organisms (Lakshmanan et al., 2021). Furthermore, acoustic biosensors were also applied in the single nucleotide polymorphism (SNP) genotyping of *Anopheles gambiae* (Papadakis et al., 2013).

Surface acoustic wave (SAW) biosensor is a type of piezoelectric biosensor. SAW devices depend on the excitation of a special acoustic mode, in which the acoustic energy is confined very near the planar surface of a solid medium (Vellekoop, 1997). SAW devices limit the energy near the surface, so the SAW sensor is highly sensitive to surface adsorption (Fogel et al., 2016). Liu et al. (Liu et al., 2015) combined SAW biosensor with graphene oxide (GO) for simple and sensitive detection of CYP2D6*10 gene polymorphism in clinical samples. In this method, the negatively charged GO was coupled to the surface of SAW chip by electrostatic interaction, and the GO modified SAW biosensor was prepared. Then the probe was fixed on the surface of GO and the mutation of DNA was detected by hybridization. Hybridization with different target analytes will produce different quality and conformational changes on the chip surface, resulting in real-time generation of different SAW signals to detect CYP2D6*10 gene polymorphism.

Thermal biosensors represent another interesting area of biosensor research and development. Biosensor platform based on change in the thermal interface conductance has been described recently (Khorshid et al., 2021). This biosensor has been proposed to be used in various applications, including mutation analysis and aptamer-based analyte detection (Khorshid et al., 2021).

The various biosensors mentioned in this review can be combined with different signal amplification technologies. Zhu et al. (Zhu et al., 2009) designed a novel mutation allele specific amplification (MASA) and electrochemiluminescence (ECL) method for the detection of point mutations in clinical samples. MASA is a mutation specific primer, which can selectively amplify the corresponding mutant alleles. The product of MASA was captured on the streptavidin magnetic beads by biotin streptavidin conjugation and detected by measuring the ECL emission of the marker. This method can detect wild type *K-ras* mutants. Biosensors have the advantages of low cost, easy manipulation and fast detection speed. In the future, biosensors can be used to detect other point mutations which has important implications in the prevention, monitoring and treatment of various diseases.

## 5 Detection of Point Mutations in Viruses by Biosensors

Due to the rapid transmission and adaptability, viruses, such as the human immunodeficiency virus (HIV) (Kaye et al., 1992), the hepatitis B virus (HBV) (Kinoshita et al., 1994; Scheiblauer et al., 2006; Hirzel et al., 2015) and the highly pathogenic avian influenza virus (HPAI) (Li et al., 2017), can cause serious life-threatening infections and lead to pandemics (Patel et al., 2019). The diffusion process of variation in populations is driven by complex interactions between the evolution of the host’s genome and the spread of the virus (Ramazzotti et al., 2020). Mutations or recombination events, which improve the adaptability of the viruses can lead to novel clinical symptoms and pandemics. For instance, HPAI influenza virus is highly prone to mutations and H5N1 and H7N9 are its typical strains (Li et al., 2017; Wang et al., 2020). Early identification and monitoring of viruses and of the emerging point mutations in their genomes is therefore important for the prevention of pandemics.

Traditional virus detection methods include virus isolation (Zhou et al., 2014), immunoassay (Ramazzotti et al., 2020) and molecular biological methods (Payungporn et al., 2004). But the novel approaches for the detection of virus mutations are based on biosensors. In this research area, the application of nano materials has been widely studied. Similar to biosensors for detecting other point mutations, a biologically bound variant of the virus aptamer is first fixed with a microprobe. Then the virus is captured, thus generating the signal output. Development of both electrochemical and optical biosensors for virus detection has been reported (Li et al., 2011; Krejcova et al., 2013). To improve virus detection, nanomaterials with excellent chemical and biological properties serving as the identification components of biosensors were developed and microfluidic technology was used to enhance the specificity and sensitivity of biosensors (Li et al., 2011; Zhang et al., 2018).

### 5.1 Biosensors for Detecting Point Mutations in SARS-CoV-2

The mutation rate of RNA viruses, related to the toxicity regulation, evolution and transmission, is very high (Pachetti et al., 2020). The mutation of viral genome depends on the viral and host enzymes involved in replication of nucleic acids. The mutation rate is affected by the template sequence and the mechanism of virus replication (Pachetti et al., 2020).

The currently ongoing COVID-19 pandemic caused by SARS-CoV-2 affects the whole world. Small mutations were detected in hosts infected with the same virus lineage (Ramazzotti et al., 2020). SARS-CoV-2 has also undergone a number of mutations to better adapt to its host.

The most common mutation detected in SARS-CoV-2, which replaced the original strain and spread quickly around the globe is the substitution of aspartate to glycine at the amino acid position 614 (D614G). D614G mutation has been suggested to be involved in a number of SARS-CoV-2 phenotypes ranging from higher transmissibility to anosmia in the affected patients (von Bartheld et al., 2021). Other mutations commonly associated with D614G mutation include the substitution of the cytosine to thymine in 5′UTR at position 241, the silent cytosine to thymine mutation at position 3,037 and the cytosine to thymine mutation at position 14,408, leading to amino acid changes in RNA dependent RNA polymerase (RdRp P323L) (Korber et al., 2020).

Other SARS-CoV-2 mutations of current interest include the substitution of asparagine by tyrosine at position 501 (N501Y), substitution of glutamate by lysine or glutamine at position 484 (E484K and E484Q, respectively), substitution of proline by histidine at position 681 (P681H) and substitution of leucine by arginine at position 452 (L452R) and others (Ramesh et al., 2021).

Notably, P681H mutation was shown to be not associated with higher infectivity or transmissibility and SARS-CoV-2 harboring P681H mutation was neutralized by sera from vaccinated hosts (Zuckerman et al., 2021). However, mutations N501Y, L452R, E484K and E484Q were shown to be associated with increased transmissibility and potentially also with higher resistance of SARS-CoV-2 to neutralizing antibodies (Wang et al., 2021a; Yi et al., 2021; Zhao et al., 2021).

Recent studies suggest that particularly the point mutations in the receptor-binding domain (RBD) of SARS-CoV-2 Spike (S) protein have the greatest impact on the infectivity and transmissibility of the virus (Barton et al., 2021; Ramesh et al., 2021). As shown above, some point mutations already circulating in the population and novel point mutations which might occur in the future can even increase resistance of SARS-CoV-2 to neutralizing antibodies (Gobeil et al., 2021). However, the level by which the resistance to neutralizing antibodies increases in SARS-CoV-2 point mutants will require further investigation as another study demonstrated that SARS-CoV-2 with variant S protein showed only low decrease in antibody neutralization (Tada et al., 2021).

Novel biosensors for the detection of the SARS-CoV-2 point mutations can contribute to the control of the pandemic. A number of biosensing technologies have been suggested for the detection of SARS-CoV-2 D614G mutation, including technologies based on SPR, antibodies, aptamers and nanobodies, Loop-mediated isothermal amplification (LAMP), and CRISPR/Cas (Zhang et al., 2021). Biosensors based on SPR, CRISPR/Cas, LAMP and aptamers have been also proposed for the detection of other SARS-CoV-2 point mutations (Xi et al., 2021). Other approaches developed recently for detecting SARS-CoV-2 point mutations include a toolset for qPCR-based SNP detection (Noerz et al., 2021) and full genome tiling array that can analyze the whole SARS-CoV-2 genome at single nucleotide resolution (Jiang et al., 2021). Dual synthetic mismatches CRISPR/Cas12a (dsmCRISPR) method for the detection of D614G mutation with high specificity and sensitivity has been presented recently (Huang et al., 2021). D614G mutation was also detected with the upgraded *Pyrococcus furiosus* Argonaute (PfAgo) mediated nucleic detection method recently (Wang et al., 2021b). Furthermore, *Francisella novicida* Cas9 (FnCas9)- based CRISPR-based method has been shown to reliably detect N501Y, E484K and T716I mutations and can be adapted also for the detection of other SARS-CoV-2 mutations (Kumar et al., 2021).

These technologies can be used for the detection of SARS-CoV-2 point-mutations and therefore can be applied in the novel biosensors for the detection of SARS-CoV-2 point mutations.

## 6 Discussion

Point mutations are directly related to the development of cancers and infectious diseases. Detection of point mutations is therefore important for the prevention and treatment of the disease. In this review, we summarized the point mutation detection methods and the application of biosensors for point mutation detection. Biosensors can overcome the disadvantages of traditional methods, such as complexity, high cost and requirement of specialized instruments. Biosensors can provide a large-scale, low-cost gene mutation screening and detection platform which meets the accuracy and real-time requirements of gene diagnosis. They can be used for personalized patient treatment. Biosensors can detect the hybridization between complementary or mismatched probes on the sensing surface by optical and electrical techniques.

The sensitivity of the sensor can be improved by combining the point mutation detection strategy with the signal amplification strategy. Although gene sequencing can accurately identify mutations, its application is limited due to high costs and technical requirements Therefore, biosensors can be used to detect point mutations in a broad-spectrum of cancers and viruses, including SARS-CoV-2. Furthermore, the combination of biosensors and signal amplification strategy is important for the investigation of point mutations, disease prevention and control and vaccine development.
